# Gold Nanoparticles Synthesized with Triple-Negative Breast Cancer Cell Lysate Enhance Antitumoral Immunity: A Novel Synthesis Method

**DOI:** 10.3390/ph18030330

**Published:** 2025-02-26

**Authors:** Raúl Rangel-López, Moisés Ármides Franco-Molina, Cristina Rodríguez-Padilla, Diana Ginette Zárate-Triviño

**Affiliations:** Universidad Autónoma de Nuevo León, Facultad de Ciencias Biológicas, Laboratorio de Inmunología y Virología, San Nicolás de los Garza 66455, Mexico; jrangelraul95@gmail.com (R.R.-L.); moyfranco@gmail.com (M.Á.F.-M.); cristina.rodriguezpd@uanl.edu.mx (C.R.-P.)

**Keywords:** antigen presentation, breast cancer immunotherapy, gold nanoparticles, immune system activation, tumor cell lysates

## Abstract

**Background:** Gold nanoparticles enhance immunity, promotes antigen uptake by antigen-presenting cells (APCs), and boost the response against tumor antigens; therefore, they are a promising delivery vehicle. Tumor lysates have shown favorable responses as inductors of anti-cancer immunity, but the effectiveness of these treatments could be improved. Hybrid nanosystems gold nanoparticles with biomolecules have been show promising alternative on uptake, activation and response on immune system. **Objectives:** This study’s objective was to develop a method of synthesizing gold nanoparticles employing a triple-negative breast cancer (4T1) cell lysate (AuLtNps) as a reducing agent to increase immunogenicity against breast cancer cells. **Methods:** Nanoparticle formation, size, and ζ potential were confirmed by surface plasmon resonance, dynamic light scattering, and transmission electron microscopy. Protein concentration was quantified using a Pierce BCA assay. The cytotoxic effects of treatments on murine macrophages were assessed, along with nanoparticle and tumor lysate uptake via epifluorescence microscopy. Using a murine model, cytokine secretion profiles were determined, and the efficacy in inhibiting the implantation of a 4T1 model was evaluated. **Results/Conclusions:** AuLtNps exhibited higher protein content than tumor lysate alone, leading to increased uptake and phagocytosis in murine macrophages, as confirmed by epifluorescence microscopy. Cytokine secretion analysis showed a proinflammatory response, with increased CD8+ and CD22+ lymphocytes and upregulation of APC markers (CD14, CD80, CD86, and MHC II+). Splenocytes demonstrated specific lysis of up to 40% against 4T1 tumor cells. In a murine model, AuLtNPs effectively inhibited tumor implantation, achieving an improved 90-days survival rate, highlighting their potential as an immunotherapy for triple-negative breast cancer.

## 1. Introduction

Cancer is a serious health problem, not only due to the disease itself but also because of the difficulty and high cost of treating it. One of the most relevant types of cancer is breast cancer; numerous therapies have been developed to reduce the size and aggression of such tumors [1,2]. One persistent concern is tumor regression or recurrence, which is the reappearance of the tumor after conventional treatments. In breast cancer, tumor recurrence is associated with the highest mortality rates [3].

To address this, new cancer treatments have been directed not only at combating the progression of the disease but also at preventing tumor recurrence [4]. Cancer immunotherapy has emerged as an alternative to treat various types of cancer [5]. Treatments include cellular therapy, which involves replacing damaged or non-functional patient cells with cells capable of fighting the disease [6]; monoclonal antibody therapy, which uses specific antibodies against molecules present in tumor cells to inhibit their development [7]; and cancer vaccines that aim to activate the antitumor immune system for long-term immunity [8]. Another approach, known as immunomodulation, involves the use of cytokines related to favorable prognosis [9].

Over the past 20 years, immunomodulation therapeutics have focused on enhancing the activity of cells responsible specifically or nonspecifically for antitumor immunity, such as natural killer cells or effector T lymphocytes [10]. However, challenges still exist in bridging the gap between innate and adaptive immunity, particularly in antigen presentation [11]. A successful immunotherapy must be able to achieve an adaptive immune response, which involves the recognition of tumor-associated antigens or neoantigens—proteins or peptides expressed exclusively by tumor cells [12].

Efforts have been made to develop vaccines against these antigens. Although they show efficacy in vivo and in vitro, many of these vaccines have not reached viable clinical applications [13]. Despite these challenges, significant progress has been made in recent years using pulsed dendritic cell therapies [14] and applying neoantigen vaccines against certain types of cancer. Notable successes include vaccines against human papillomavirus preventing cervical cancer and the recently approved vaccine against prostate cancer [15].

An emerging alternative proposes the use of autologous tumor lysates to generate specific immunity against the type of tumor from which the lysate was derived [16]; this is often combined with dendritic cell therapies to obtain a stronger response from the immune system [17]. Nevertheless, the efficiency of antigen presentation using these lysates could be improved. Studies using this therapeutic approach often report low effectiveness due to antigen presentation issues [18]. This happens because the same antigen-presenting cells responsible for presenting antigenic variants to T lymphocytes face difficulties in inducing specific antigen recognition and promoting antigen-specific cell lysis.

One of the initial methods developed to enhance the antigen capture processes from tumor cells and even whole tumor cells uses polymeric nanoparticles [19]. These nanoparticles encapsulate antigenic fragments derived from patient tumor cells, employing a polymer such as polylactic acid [20]; the encapsulation process includes antigens associated with autologous tumors. This approach put forward a therapy based on dendritic cells to stimulate autologous CD8+ T cells [21]. Through clinical trials, it was possible to increase the populations of antitumor CD8+ T lymphocytes, in addition to enhancing the secretion of IFN-γ and reducing the levels of IL-10 [22]. This effect was observed specifically in the group of patients treated with polymeric nanoparticles loaded with tumor lysate [23].

In recent years, nanotechnology has been used to deliver substances into biological systems, including tumor-associated antigens and neoantigens [24,25]. Gold nanoparticles, when attached to these molecules, can specifically activate the immune system, even generating antigen-specific antibodies [26,27].

This study aims to combine tumor lysates with gold nanoparticles, developing a one-step synthesis using a gold precursor salt and tumor lysates from a murine triple-negative breast cancer cell line (4T1). The goal is to enhance a specific antitumor immune response against this cell line using a murine model sensitive to tumor implantation.

## 2. Results

### 2.1. Formation of Gold Nanoparticles

**Figure 1:**
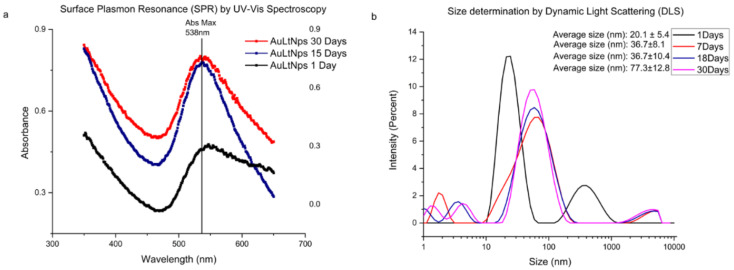
Characterization of gold nanoparticles synthesized from tumor lysate derived from triple-negative murine breast cancer (4T1). (**a**) Characterization through the identification of the surface plasmon resonance of AuLtNps up to 30 days. (**b**) Particle size determination by DLS.

When measured by UV-VIS spectroscopy, metal exhibits a characteristic band on a nanometric scale known as Surface Plasmon Resonance (SPR). In the case of gold, this band is within the range of 510 to 550 nm. Gold nanoparticles synthesized from tumor lysate derived from triple-negative murine breast cancer (AuLtNps) had a maximum absorbance at 537 nm, which remained consistent from day 1, with an intensity of 0.6 relative units (RU), until reaching 0.9 RU on day 30 (Figure 1a). AuLtNps showed a stable size measured by Dynamic Light Scattering (DLS), starting at 20 nm on day 1, increasing to 36.72 nm on day 7 through day 18, and reaching a size of 77.39 nm on day 31, indicating that the size increase is time-dependent (Figure 1b).

### 2.2. Determination of ζ Potential

**Figure 2:**
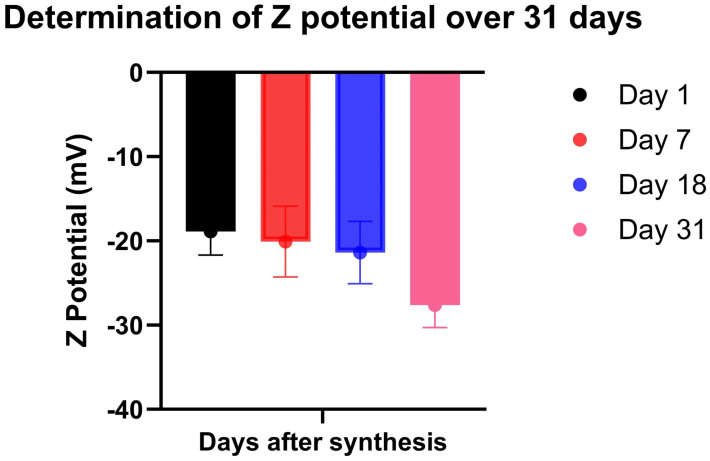
Determination of ζ potential up to 30 days post synthesis.

The electric superficial charge, known as ζ potential, was determined through DLS on days 1, 7, 18, and 31 to assess the particle stability in relation to the surrounding medium. A time-dependent increase in the surface charge of AuLtNps was identified, starting with a net charge of −18.9 mV on day 1, −20.1 mV on day 7, and −21.4 mV on day 18, eventually reaching −27.6 on day 31 (Table 1, Figure 2), indicating an increase in the repulsive force between particles.

### 2.3. Morphology Analysis of Gold Nanoparticles

**Figure 3:**
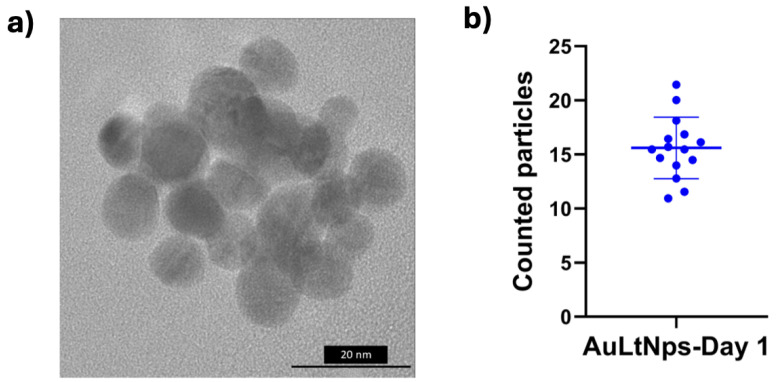
(**a**) Transmission electron microscopic image on the 1st day of AuLtNps synthesis. (**b**) Graphic representation of counted particles from the TEM image.

Transmission Electron Microscopy (TEM) was used to observe the morphology and size of the nanoparticles on the first day of synthesis. The spherical morphology characteristic of metallic core–shell particles was observed (Figure 3a). Particle size analysis was conducted, revealing that most particles fell within the range of 9 to 16 nm (Figure 3b).

### 2.4. Determination of Protein Concentration in AuLtNPs

**Figure 4:**
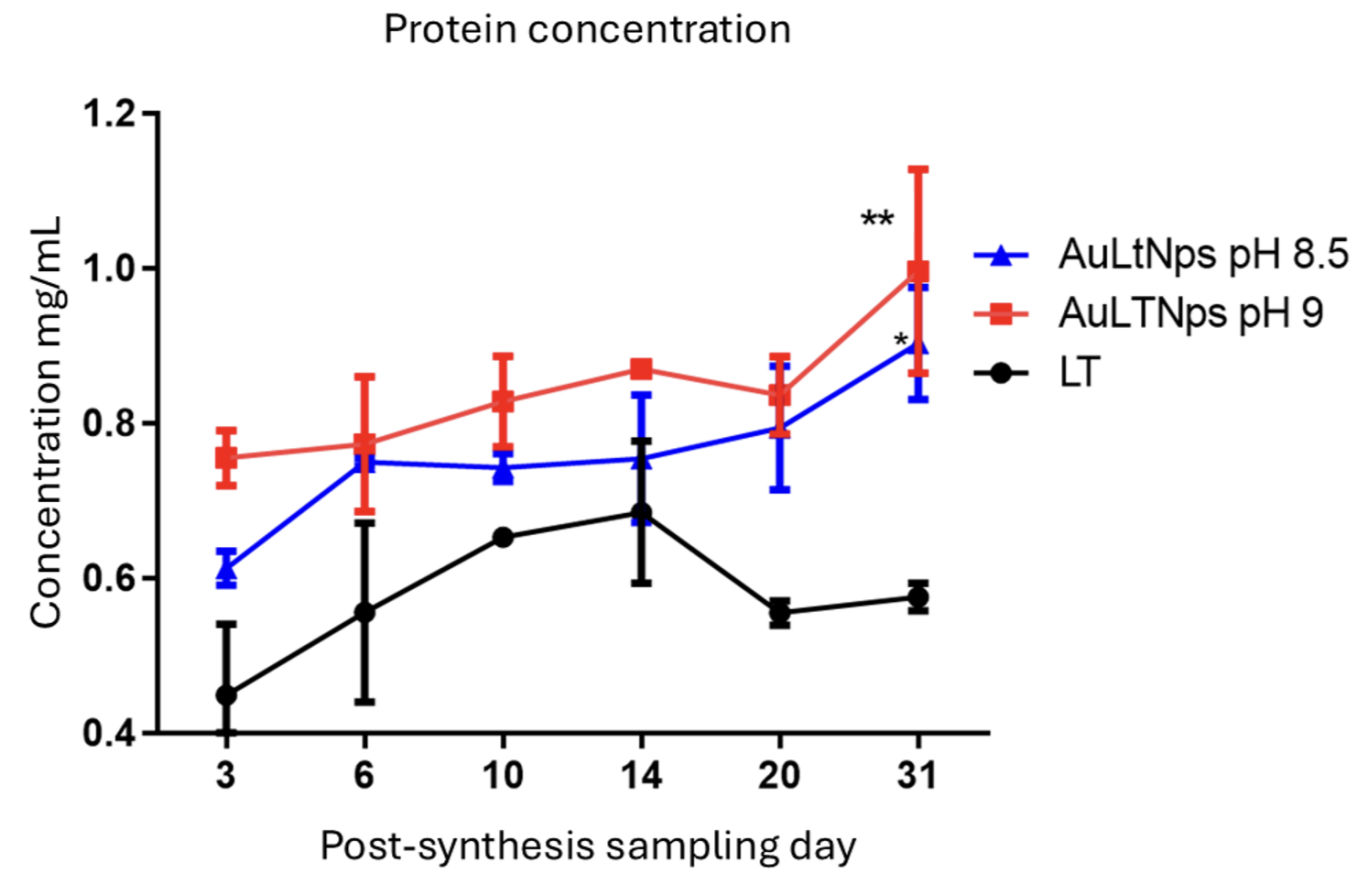
Determination of protein concentration in AuLtNPs. A significant difference was observed between AuLtNps pH 8.5 and AuLTNps pH 9, which was more pronounced at day 31 of evaluation compared to the control. * *p* < 0.05 and ** *p* < 0.01.

In order to confirm that the time-dependent growth of nanoparticles is associated with the total protein concentration, two nanoparticle solutions were selected with pH values of 8.5 and 9, using Tumor Lysate (LT) as control. A significant difference was observed between AuLTNps with pH 8.5 and AuLtNps with pH 9, which was more pronounced on the 31st day of evaluation compared to the control (Figure 4); this could be related to the degradation of tumor lysate and suggests that a protein is attached on the nanoparticles surface.

Polymeric nanoparticle systems have been successful in controlled molecular release [28]. Although, initially, the protein uptake phenomenon might be considered unfavorable, it proves to be advantageous in terms of antigen presentation. In comparison, gold nanoparticles have been proven to concentrate the antigen, allowing for the absorption of more antigenic variants confined within a nanoparticle [29] and a broader immune response against antigens.

The biological activity of gold nanoparticles is significantly influenced by factors such as size and reducing agent. A novel method for gold nanoparticle synthesis was developed in [30], where tumor lysate was used as the reducing agent and HAuCl_4_ was used as the metal precursor.

Repeated intraperitoneal administration of 12.5 nm AuNps did not elicit significant oxidative stress in mice. Given that nanoparticle size affects the biological effects in a model, smaller particles induced transient hepatic changes, whereas larger particles accumulated in the spleen. These findings highlight the importance of particle size in determining biodistribution and toxicity, indicating a dose-dependent accumulation pattern without associated physiological impairment [31,32].

It was determined that the size and negative surface charge increase over time, reaching 77 nm and −27.6 mV, respectively, after 30 days of synthesis, contributing to their stability. This increment in both size and surface charge is attributed to the protein corona effect. This phenomenon involves the net charge of the particle attracting proteins with an opposite charge. It has been observed that AuLtNps captures proteins from the surrounding solution, forming a hard and soft protein layer on the surface [33,34].

Although these interactions are not permanent, if the initial conditions are maintained, the charge present on the particle remains stable [35,36]. This phenomenon explains the increase in protein concentration over time, even without adding more protein to the sample; a similar phenomenon was reported on concentration of DNA or RNA with a cationic polymer [37,38].

### 2.5. Pearson Correlation Analysis

Pearson correlation of AuLtNps is a statistical method that measures the similarity or correlation between SPR intensity, particle size, surface charge, and protein concentration (Table 1) by comparing their attributes and calculating a score ranging from −1 to +1 (Table 2). A high score indicates high similarity and a high correlation between particle size and ζ potential. As time passes, the particle size and ζ potential increase, which indicates the stabilization of the components in the synthesis [39].

### 2.6. Cytotoxicity Analysis

**Figure 5:**
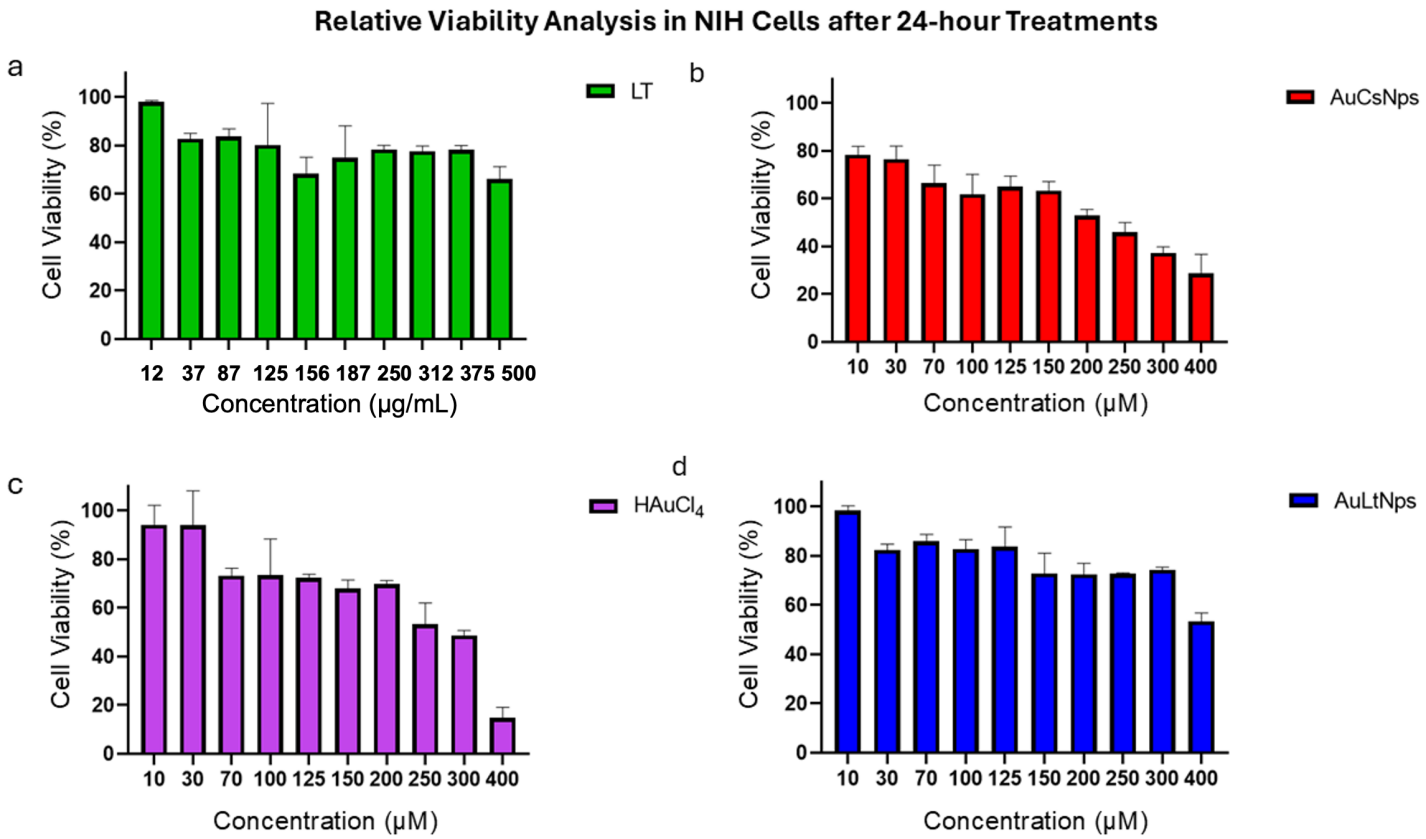
Relative viability analysis by MTS in NIH cells after 24 h. (**a**) LT is shown in green, (**b**) AuCsNps in red, (**c**) HAuCl_4_ in purple, and (**d**) AuLtNps in blue. On AuLtNps, the cell viability remained at 80%, even at concentrations as high as 300 μM.

In order to determine the dose of AuLtNps and the controls, a dose-dependent cytotoxicity evaluation was performed on non-cancerous cell line NiH-3T3. On AuLtNps, the cell viability remained high, even at concentrations as high as 300 μM (Figure 5d).

AuLtNps shows a minor cytotoxic effect on the fibroblast cell line in comparison to AuCsNps (Figure 5b) and HAuCl_4_. This is due to the protein corona effect of proteins of the tumoral lysate on AuLtNps and the lower effect of ion release from gold atoms, in contrast to the AuCsNps and HAuCl_4_, which lack protein in the synthesis [40].

Two treatments were administered at equivalent volumes (1:1 v/v), HAuCl_4_ concentrations are expressed in micromolar units (μM), whereas those of LT are expressed in μg/mL; the difference in units reflects specific experimental considerations.

### 2.7. Macrophage Uptake

**Figure 6:**
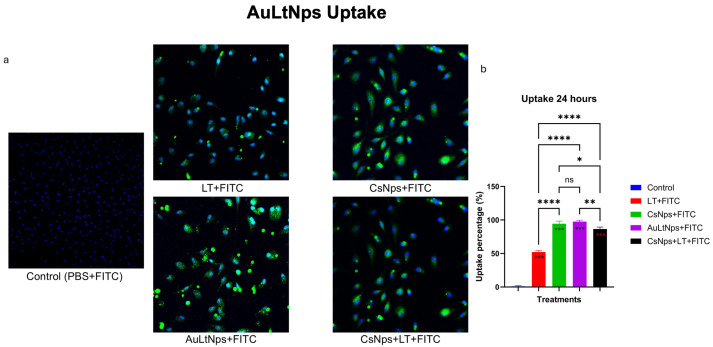
Determination of AuLtNps uptake in mouse intraperitoneal macrophages after 24 h. (**a**) Control group, tumor lysate (LT+FITC), AuCsNps (AuCsNps+FITC), AuLT (AuLtNps+FITC), and AuCsNps+LT(AuCsNps+LT+FITC). All cells were stained with blue DAPI to mark the nucleus and FITC as bright-green fluorescence. (**b**) Graph representing fluorescence detection. * *p* < 0.05, ** *p* < 0.01, and ***/**** *p* < 0.001, ns, not significant.

To determine whether AuLtNps were taken up by murine macrophages, the cells were extracted and cultured. Fluorescence was detected inside the macrophage, confirming the antigen uptake was more efficient for AuLtNps compared with the control LT (Figure 6).

The subcutaneous immunization route employed in this study is well-established, thanks to its minimal organ toxicity and bioaccumulation. Subcutaneously injected gold nanoparticles are efficiently phagocytosed by macrophages, which subsequently migrate to lymph nodes and blood vessels. This suggests that the reticuloendothelial system plays a pivotal role in AuNps clearance. Tumor lysate is normally taken up by intraperitoneal macrophages [41,42]; however, functionalization with gold nanoparticles potentiated the effect, as seen on the AuLtNps and AuCsNps samples (Figure 6a). The combination of CsNps+LT resulted in lower uptake compared to AuLtNps.

Fluorescence microscopy revealed interference within cells treated with AuLtNps. This interference did not occur in the case of AuCsNps but did with LT, suggesting distortion or interference as a signal that originates from inside the cell and not from the cell surface. Additionally, the overlap of DAPI staining for nuclei combined with the green fluorescence resulted in the cyan color observed in cells treated with LT and AuLtNps but not with CsNps, aligning with findings reported by Gao-Na Shi [19].

### 2.8. Cytokine Secretion Analysis

**Figure 7:**
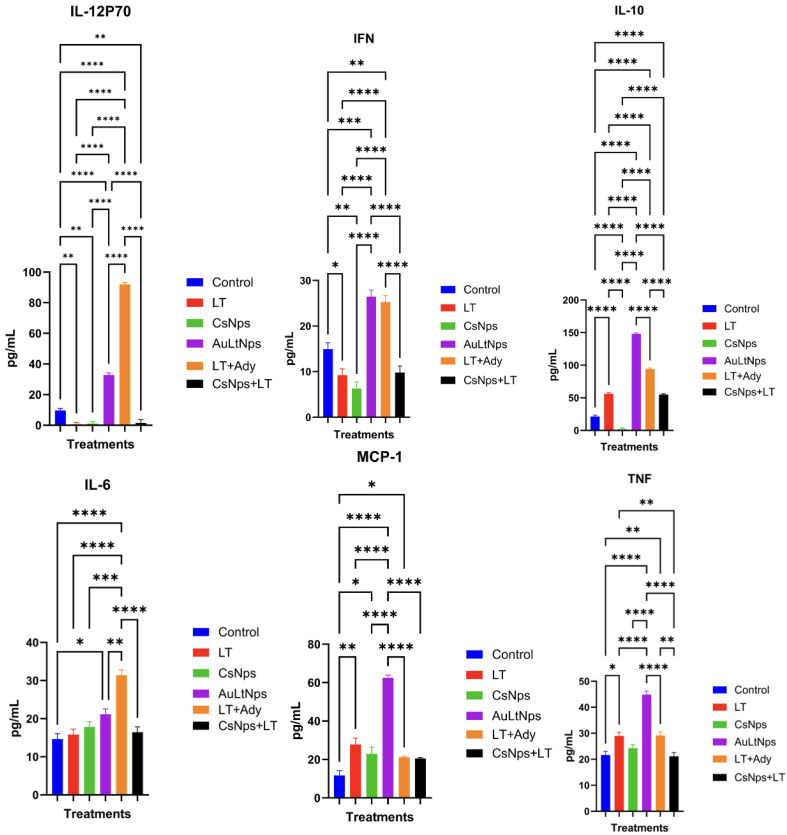
Determination of proinflammatory cytokines in mice sera 24 h after the second immunization. Experimental groups: control, tumor lysate, CsNps, tumor lysate + adjuvant [Al(OH)_3_], and AuCs + tumor lysate. Mice treated with AuLtNps showed increments of IFN, IL-10, MCP-1, IL-6, and TNF. A statistical ANOVA with mean comparison was conducted to determine differences between groups. * *p* < 0.05, ** *p* < 0.01, and ***/**** *p* < 0.001.

**Figure 8:**
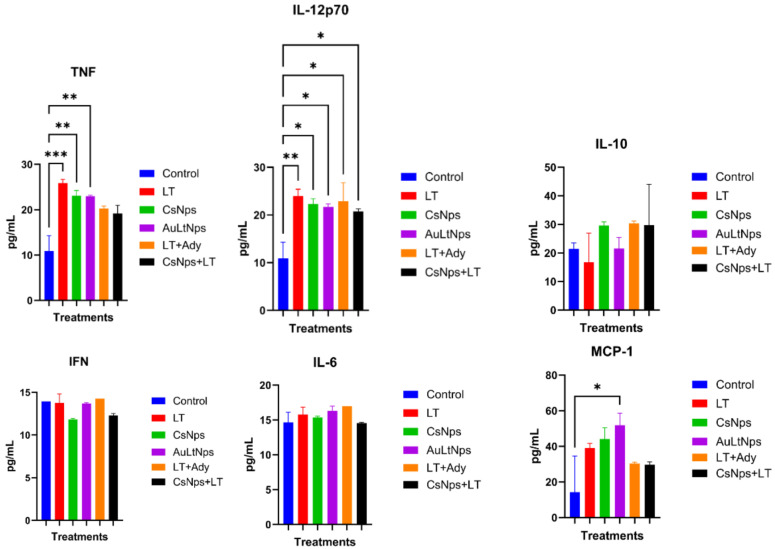
Determination of the presence of proinflammatory cytokines in mouse sera 7 days after immunization. Experimental groups: control, tumor lysate, CsNps, tumor lysate + adjuvant [Al(OH)_3_], and AuCs + tumor lysate. The secretion of IL-10, IFN, and IL-6 was normalized; however TNF, Il12p70, and MCP-1 were elevated compared with the control in mice treated with AuLtNps, LT, and CsNps. A statistical ANOVA with mean comparison was conducted to determine differences between groups. * *p* < 0.05, ** *p* < 0.01, and *** *p* < 0.001.

Mice immunized with AuLtNps and LT+Ady exhibited increased secretion of proinflammatory cytokines (IFN, TNF, IL-6, IL-10, and IL-12p70), whereas a high level of secretion of MCP-1 was observed in the group treated solely with AuLtNps (Figure 7). To determine whether prolonged exposure to different treatments induced chronic inflammation, sera were analyzed one week after the last immunization. Secretion of IL-6, IL-10, and IFN showed no variation compared to the control (Figure 8), whereas TNF, IL-12p70, and MCP-1 displayed variations relative to the control. Additionally, there was a noticeable increase in the secretion of IL-12p70 in all treatment groups except the control group.

Mice immunized with AuLtNps exhibited an increase in the secretion of proinflammatory cytokines such as IFN, IL-12p70, TNF, and MCP-1 after the second immunization compared to the other treatment groups. This demonstrates the potential of AuLtNps to activate the immune system. TNF secreted by macrophages is crucial for the formation and maintenance of granulomas, which play a critical role in the defense against intracellular organisms. TNF also participates in leukocyte trafficking and immune complex elimination [43].

IL-12p70 secretion, primarily produced by antigen-presenting cells (APCs), is crucial in both cellular and humoral immunity, as it induces interferon production and activates CD4+ T cells with a lymphocyte t helper 1 (Th1) phenotype. IL-12p70 also plays a significant role in T-cell trafficking, inducing functional adhesion molecules, such as selectin P and E ligand, which are recruited to sites requiring Th1-type immune responses [44].

A high level of MCP-1 secretion was observed in mice immunized with AuLtNps, which is key in regulating monocyte/macrophage migration and infiltration. This stimulation promoted monocyte migration to the bloodstream through the vascular endothelium, a fundamental process in immune surveillance. One week after the last immunization, IFN, IL-6, and IL-10 levels returned to normal compared to untreated controls. TNF, IL-12p70, and MCP-1 secretion showed no difference between groups, although they were higher than the control.

There was no chronic proinflammatory effect in the mice whose cytokine levels and secretion profiles were normalized after seven days compared to the untreated control. Rapid accumulation of intraperitoneally injected AuNps in the abdominal adipose tissue and liver of C57BL/6 mice was accompanied by reduced TNF-α and IL-6 mRNA. These findings suggest potential anti-inflammatory effects without overt organ damage [45]. MCP-1 was the only cytokine with a significant elevation compared to the control, indicating ongoing immunogenic surveillance by macrophages and antigen-presenting cells, even after seven days [46].

### 2.9. Lymphoid Lineage Activation and Antigen-Presenting Cell Markers

Increases in the proportions of CD3, CD8, and CD22 cells were observed, whereas the proportion of CD34+ cells remained constant across treatments. A significant increase in CD3+ cells was observed for all treatments, except for the LT group.

**Figure 9:**
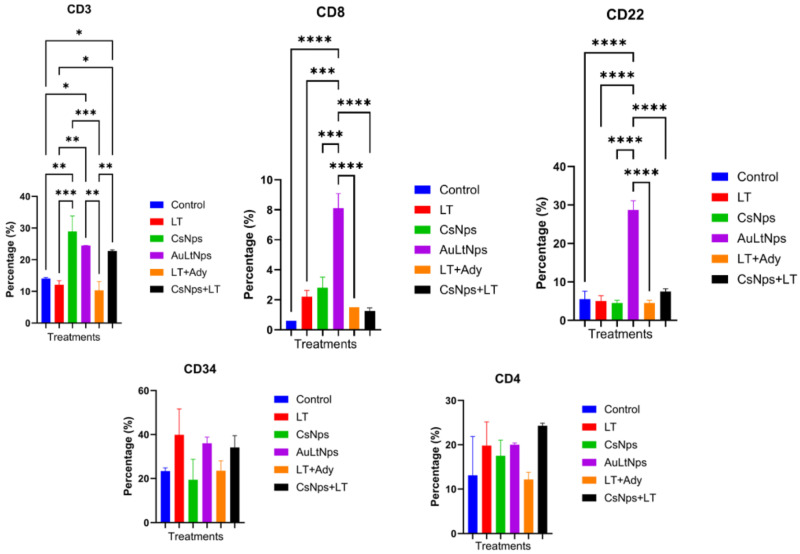
Determination of differentiation markers CD8, CD4, CD3, CD22, and CD34 in mouse spleen 7 days after immunization. Experimental groups: control, tumor lysate, CsNps, tumor lysate + adjuvant [Al(OH)_3_], and AuCs + tumor lysate. Increments of CD3, CD4, and CD8 markers on cells were used on mice treated with AuLtNps, suggesting the presence of cytotoxic T lymphocytes and an increase in mature B lymphocytes. A statistical ANOVA was conducted to determine differences between the groups. * *p* < 0.05, ** *p* < 0.01, and ***/**** *p* < 0.001.

The proportion of CD8+ cells showed a highly significant difference between AuLtNps and all other groups. This also occurred with CD22+, where the proportion of positive cells increased by up to three times when compared with the other treatments (Figure 9).

Markers for APCs are anti-CD80, CD11c, CD11b, MHC-II, and CD14. A highly significant increase in CD80 expression was identified in the CsNps, AuLtNps, LT+Ady, and CsNps+LT groups compared to the control. For CD86 cells, there was only a significant difference in the group treated with AuLtNps. In the case of CD11c+ and CD11b+ cell proportions, no significant differences were found among the treatments, although there was some variation across all groups.

**Figure 10:**
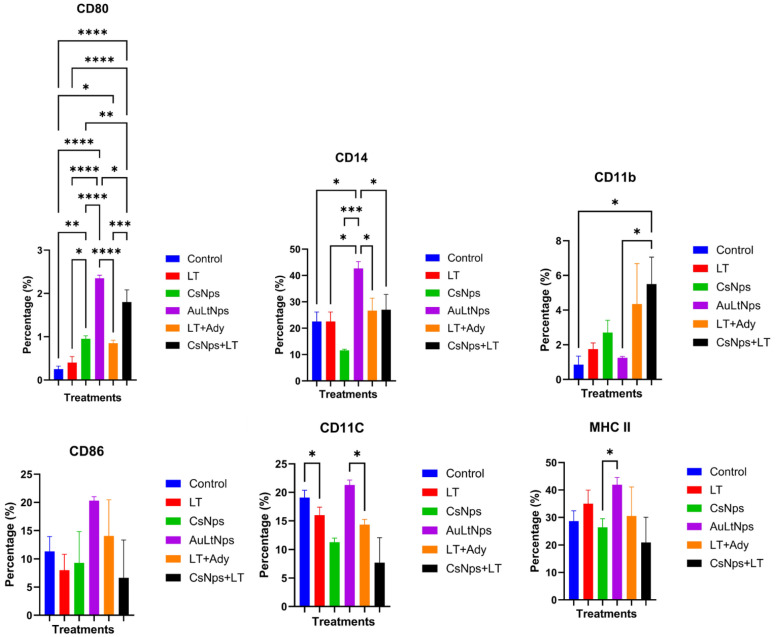
Determination of differentiation markers CD80, CD14, CD11c, CD11b, CD86, and MHC II in mice lymph nodes 7 days after immunization. Experimental groups were euthanized: Control, Tumor Lysate, CsNps, Tumor Lysate + Adjuvant [Al(OH)_3_], and AuCs + Tumor Lysate. An increase of cells CD80, CD14, CD86, CD11c and MHC II in the mice treated with AuLtNps, which suggests a favorable maturation process for dendritic cells in lymph nodes. A statistical ANOVA was conducted to determine differences between the groups. * *p* < 0.05, ** *p* < 0.01, and ***/**** *p* < 0.001.

Cells marked positive for MHC II and CD14+ showed a significant increase in the AuLtNps group in comparison with CsNps which displayed a notable decrease of this marker when compared to the control (Figure 10) [47].

### 2.10. Specific Lysis Activity

**Figure 11:**
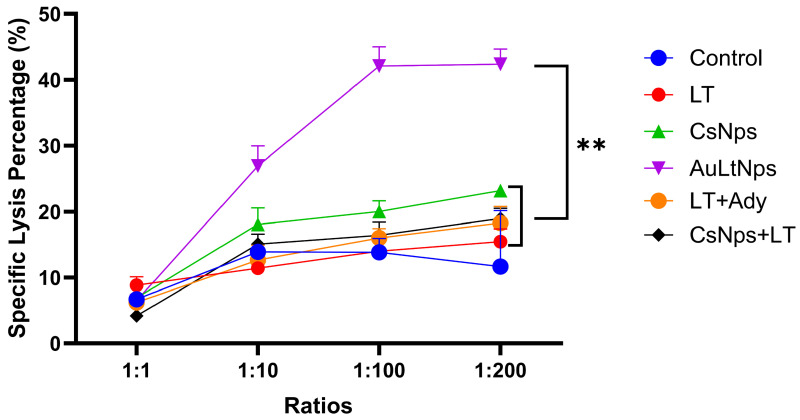
Assessment of prophylactic efficiency in a murine model of triple-negative breast cancer. Evaluation of specific cytotoxicity in lymphocytes extracted from the spleens of immunized mice at different ratios showed that cells treated with AuLtNps exhibited specific lysis of lymphocytes against 4T1 cells. A statistical ANOVA was performed to determine differences between the groups. ** *p* < 0.01.

Primed splenocytes of previously immunized mice exhibit an immune response against cancer cells due to their specific lytic capacity. To evaluate this, splenocytes extracted from immunized mice were cultured at ratios of 1:1, 1:10, 1:100, and 1:200 (4T1: splenocytes) and incubated for 24 h with viability marker 7-AAD, which is a DNA intercalator that fluoresces under flow cytometry only in dead or damaged cells. Only the AuLtNp group exhibited a significant difference, where the percentage of specific lysis reached up to 40% at ratios of 1:100 and 1:200 (Figure 11).

Splenocytes from immunized mice can specifically recognize 4T1 breast cancer cells, inducing an immunological defense. Splenocytes bind to cancer cells, activating the lytic capacity of the lymphocytes, which releases granzymes and perforins that produce pores in the cell membrane. Cell death is measured by the 7AAD marker, which enters dead cells. In controls, the 7AAD marker could not enter the impermeable cell membrane, indicating that AuLtNps showed a highly significant difference in comparison to all controls and that the immunization process of gold nanoparticle synthesis with tumoral lysates primes splenocytes to recognize 4T1 breast cancer cells [48].

### 2.11. Antitumor Efficacy of AuLtNps in Previously Immunized Balb/C Mice

A tumor challenge was conducted by inoculating 5 × 10^5^ viable 4T1 cells into the mammary area of mice using an orthotopic model. The day when the tumor became palpable in the inoculated area was recorded as the tumor implantation day.

**Figure 12:**
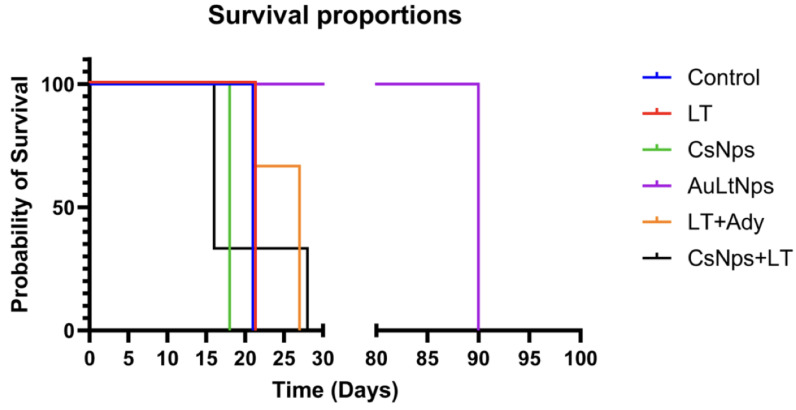
Comparative survival table for each experimental group extended up to a maximum of 90 days. Mice treated with CsNps had a maximum survival of 28 days, and with AuLtNps, it was possible to extend the survival rate to day 90.

The AuLtNps group did not exhibit tumor implantation, with only slight inflammation at the injection site on the second day. In the LT group, three mice had tumor implantation on day 7, whereas one had it on day 10. The CsNps and LT+Ady groups showed tumor implantation on day 6, whereas the CsNps+LT group displayed tumor implantation on day 10. In the control group, tumor implantation occurred in all mice on day 7. All experimental groups were observed for a minimum of 21 days, without significant weight changes, but some groups experienced noticeable health deterioration (Figure 12).

On day 21, all individuals in the control group, as well as the LT group, were ethically euthanized due to the presence of metastases. In the CsNps group, one mouse died on day 16, and the remaining three were sacrificed on day 28. In the LT+Ady group, only two mice survived until day 27, whereas one died on day 5 and another on day 11. The CsNps+LT group was sacrificed on day 18 due overall deterioration. All four individuals treated with AuLtNps survived over 90 days without showing signs of tumor occurrence or metastasis.

The control group had an average lifespan of 21 days, whereas the LT group had an average lifespan of 20.5 days. The CsNps group had an average lifespan of 16 days, whereas the the CsNps+LT and LT+Ady groups reached 28 and 27 days, respectively. Finally, the AuLtNps group had an average lifespan of over 90 days (Table 3; Figure 12).

In vitro studies have shown the co-stimulatory effects of IL-10 with antigen immunization, promoting antigen-specific T-cell proliferation. An increase in IL-10 secretion is associated with proliferation of murine cytotoxic CD8+ T cells, and combined with IL-2, it enhances their cytolytic activity [49].

The anergy of CD4+ cells has been demonstrated between high IL-10 secretion and low expression of MHC II in APCs, which inhibits the MHC-CD28 stimulation pathway in T cells [50], as seen on cells treated with CsNps. In contrast, MHC II-positive cells in mice immunized with AuLtNps are increased by up to 40%, indicating activation of humoral immunity and active antigen presentation.

These results support the hypothesis that subcutaneous administration of AuNps is a safe and effective approach for inducing immune responses, as subcutaneous injection of AuNps induces localized macrophage recruitment and subsequent migration to regional lymph nodes. This mechanism likely contributes to the observed localization of nanoparticle-induced effects. Furthermore, repeated immunizations may promote a tolerogenic response, mitigating adverse effects.

Finally, mice immunized with AuLtNps showed a significant inhibition of 4T1 tumor cell implantation compared to all experimental groups, suggesting an antitumoral effect of the nanoparticles. An increase in antigen-presenting cells for the AuLtNps, AuCsNps, and LT+Ady groups correlates with a longer survival time, as does the inflammation pattern when secreting TNT, IL-12p70, and MCP-1 24 h and one week after the last immunization. AuLtNps exhibited a proinflammatory cytokine secretion profile with increases in CD3, CD8+ T, and CD4+ T cells, as well as an increase in the proportion of CD22+ cells, suggesting that nanoparticles obtained using tumor lysate as a reducing agent activate both humoral and cellular immunity.

## 3. Materials and Methods

### 3.1. Synthesis of Citrate-Reduced Gold Nanoparticles

Gold nanoparticles were synthesized based on the Turkevich method. The HAuCl_4_ (Sigma Aldrich, St. Louis, MO, USA) solution was vigorously mixed with a 1% sodium citrate (Sigma Aldrich, St. Louis, MO, USA) solution at 2 mM in a 9:1 relation v/v. The mixture was exposed to UV light in a Crosslinker Ultraviolet (UVP, Upland, CA, USA) device at an intensity of 6000 μJ/cm^2^ for 30 min. Afterwards, the solution was allowed to rest at room temperature for 24 h, then refrigerated. These nanoparticles were employed as an experimental control [51].

### 3.2. Cell Lines

Murine breast cancer cell line 4T1 and murine fibroblast cell line NIH-3T3 were maintained under standard culturing conditions at 37 °C, 85% relative humidity, and 5% CO_2_ in Dulbecco’s Modified Eagle Medium (DMEM) (Gibco, New York, NY, USA) supplemented with 10% fetal bovine serum (FBS) (Gibco, New York, NY, USA) and 1% antimycotic antibiotic (Gibco, New York, NY, USA).

### 3.3. Synthesis of Tumor Lysate-Reduced Gold Nanoparticles

Gold nanoparticles reduced from a lysate of 4T1 cells were obtained from 1,200,000 viable cells lysed by sonication in three rounds of 10 min each. The lysate was mixed vigorously for with a 2 min with a 2 mM HAuCl_4_ solution, then exposed to UV light using an Ultraviolet Crosslinker at an intensity of 6000 μJ/cm^2^ for 30 min. The solution was then allowed to cool in a refrigerator and stored for one week before use [52].

### 3.4. Characterization of Gold Nanoparticles

#### 3.4.1. Surface Plasmon Resonance

Gold nanoparticles were characterized by Surface Plasmon Resonance (SPR) using a Nanodrop 2000c (Waltham, MA, USA) with UV-VIS spectroscopy. Measurements were taken every 3 days until day 30 [53].

#### 3.4.2. Size and ζ Potential of Gold Nanoparticles

Nanoparticles were loaded in a capillary cell for spectrophotometry and analyzed at 25 °C; the size was determined by DLS. Laser Doppler electrophoresis was used to determine the ζ potential using an NS90 nanosizer instrument (Malvern Instruments, Malvern, UK) [54]. Measurements were obtained on days 1, 5, 12, 15, 24, and 30.

#### 3.4.3. Size and Morphology Analysis via Transmission Electron Microscopy

Chitosan nanoparticles were mounted uniformly on carbon adhesive tape that was attached to a sample holder and analyzed by transmission electron microscopy (TEM). The resulting file was analyzed using Gwyddion software, version 3.0 to obtain size distribution histograms [55].

#### 3.4.4. Protein Quantification Curve

The protein concentration was quantified using Pierce BCA Protein Assay Kits, (Thermo Scientific, Rockford, IL, USA) following the instructions of the manufacturer. Samples were analyzed via UV-VIS spectroscopy using a Nanodrop 2000c (Thermo Fisher, Walthman, MA, USA) and read at a wavelength of 560 nm. Measurements were performed in triplicate on days 3, 6, 10, 14, 20, and 31. The standard curve was made using albumin as a reference protein, at concentrations ranging from 0.2 to 2 mg/mL. After obtaining the data, mean variation analyses were performed using an ANOVA test for each measurement day [56].

### 3.5. Cytotoxicity Assay in NIH-3T3 Cells

Five thousand viable NIH-3T3 cells were treated for 24 h with tumor lysate-reduced gold nanoparticles (AuLtNps) at the following concentrations: 15, 35, 75, 120, 180, and 300 μM. Controls included tumor lysate, HAuCl_4_, and sodium citrate nanoparticles (CsNps). Relative cell viability was evaluated using 3-(4,5-dimethylthiazol-2-yl)-2,5-diphenyltetrazolium bromide (MTT) (Cayman Chemical, Ann Arbor, MI, USA) [57]. The absorbance data were calculated using the following formula:

(1) $$\mathrm{Percentage}\;\mathrm{of}\;\mathrm{relative}\;\mathrm{viability}=\frac{\mathrm{Treatment}\;\mathrm{absorbance}\;(\mathrm{nm})}{\mathrm{Control}\;\mathrm{absorbance}\;(\mathrm{nm})}\;\;\times \;\;100$$

### 3.6. Animal Model Immunization Schedule

Seven experimental groups were used: tumor lysate (LT), sodium citrate nanoparticles (CsNps), tumor lysate nanoparticles (AuLtNps), CsNps + LT, LT + [Al(OH)_3_], and a control group using phosphate-buffered saline (PBS). Each group consisted of six female Balb/C mice aged 6 weeks. The immunization schedule followed the protocol described by Shi [19]; mice were injected once a week for three weeks (−21, −14, and −7), with a one-week break before subsequent tests. Immunization was performed without exceeding a maximum volume of 200 μL per mouse.

### 3.7. Tumor Challenge

One week after the final immunization, a subcutaneous inoculation was performed in the mammary gland using a 1 mL syringe and a 27 G needle, (Nipro, Mechelen, Belgium) injecting 500,000 viable 4T1 cells resuspended in 50 μL of Iscove’s Modified Dulbecco’s Medium (IMDM) medium supplemented with 10% SFB. Tumor implantation and development were closely monitored, with a maximum follow-up period of 21 days after inoculation [58]. If any mice exhibited excessive pain or difficulty in feeding or moving, they were euthanized following relevant guidelines.

### 3.8. Anesthesia and Euthanasia

All the in vivo experiments were performed following Mexican norm [59]. Mice were anesthetized using a mixture of ketamine (100 mg/kg) (Vetoquinol, Escobedo, México) and xylazine (10 mg/kg) (PiSA, Jálisco, México); a response test was conducted by gently pricking one of the mouse’s paw pads. Once anesthesia was ensured, cardiac puncture was performed using a 1 mL syringe with a 25 G needle. The collected blood was stored in anticoagulant-containing tubes. After blood collection, cervical dislocation was carried out, and the mouse’s spleen and lymphatic nodules were obtained [60].

### 3.9. Peritoneal Macrophage Antigen Uptake Assay

An incision was made in the skin covering the mouse’s abdomen, and the skin was gently pulled back from the peritoneal membrane without piercing it. A total volume of 7 mL of DMEM was injected into the peritoneal cavity, gently agitated, then withdrawn from the mouse’s peritoneal cavity and centrifuged at 1350 rmp for 10 min. The obtained cells were counted, and 100,000 cells were plated and incubated for 4 days with supplemented DMEM [61].

AuLtNPs, soluble LT, PBS, and CsNps treatments were conjugated with the fluorophore FITC. This was achieved through coupling by constant agitation for 3 h, using FITC at a final concentration of 1 mg/mL. After conjugation, the treatments were independently exposed to murine intraperitoneal macrophages for 24 h. Subsequently, the cell wells were washed to remove any remaining FITC that did not enter the cell, and DAPI staining was performed to identify the presence of cell nuclei. Samples were analyzed using an epifluorescence microscope to capture images of each treatment and count how many cells exhibited the characteristic green positive staining of the FITC marker. Only cells that presented both DAPI and FITC staining in or around the nucleus were considered positive. Finally, the percentage of cells that tested positive for FITC staining per treatment was plotted.

### 3.10. Determination of Proinflammatory Cytokines

After blood extraction, the serum was separated by centrifugation and analyzed for proinflammatory cytokines by flow cytometry using a BD CBA proinflammatory cytokine kit following the manufacturer’s instructions (BD accuriTMC6, Piscataway, NJ, USA) [62].

### 3.11. Evaluation of Cellular Markers in Mouse Spleen After Immunization

After extraction of the spleen, perfusion with PBS was conducted to obtain lymphoid cells from the tissue. The cells were incubated with membrane markers CD4, CD3, CD8, CD22, and CD34 at a concentration of 1 μg/mL for 1 h, then evaluated by flow cytometry [63].

### 3.12. Identification of Macrophage Differentiation Profile in Lymph Nodes of Immunized Mice

Three mice per group from all experimental groups were euthanized on day 1 of immunization, and peripheral lymph nodes from the axillary and inguinal were extracted. Perfusion with PBS was performed, passing at least 5 mL through each lymph node until a turbid solution was obtained. The lymph node cells were counted, and a minimum of 100,000 cells was used for each assay. Membrane markers CD14, CD11c, CD11b, CD80, CD86, and MHCII were employed at a concentration of 1 μg/mL for subsequent analysis by flow cytometry [64].

### 3.13. Lymphoid Lineage Activation and Antigen-Presenting Cell Markers

One week after the final immunization (day 1), three mice from each group were euthanized, and their spleens were extracted. Perfusion with PBS was performed, passing at least 5 mL through the spleen until a turbid solution was obtained. The extracted cells were disaggregated and marked with anti-CD3, CD4, CD8, CD22, and CD34 antibodies and analyzed by flow cytometry. The population of CD3+ cells was selected; then, the presence of CD4+ and CD8+ cells was determined [64].

### 3.14. Evaluation of Specific Cytotoxicity of T Lymphocytes

After the spleen was mechanically disaggregated, the lymphoid cells from the tissue were obtained by density gradient with Ficoll. These cells were cultured and subsequently exposed to viable 4T1 cells at ratios of 1:1, 1:10, 1:100, and 1:200. The cells were cultured for 24 h, and viability marker 7-Aminoactinomycin D (7-AAD) was used to identify dead cells. Gating was performed to determine the viability of 4T1 cells recollected from a co-culture by flow cytometry [65].

### 3.15. Antitumor Effect of AuLtNps in Previously Immunized Balb/C Mice

Previously immunized groups were inoculated subcutaneously with 5 × 10^5^ viable 4T1 cells in the mammary gland. The appearance of the tumor was registered. The evaluation included implantation time, potential metastasis, and established survival [66].

### 3.16. Statistical Analysis

The data are represented as means ± standard deviation. Differences between the control group and experimental groups were analyzed using Student’s t-test, whereas differences between groups were determined using a one-way ANOVA using GraphPad 10 software.

## 4. Conclusions

A novel synthesis method was developed to obtain gold nanoparticles using lysates derived from the 4T1 tumor cell line as a reducing agent, which showed immunostimulatory properties in vitro and in vivo. This method simplifies the production process and reduces synthesis costs, as it eliminates the need for a wide range of reducing and stabilizing agents.

The prophylactic effect of AuLtNps revealed a contrasting lack of tumor implantation in a murine model that was previously immunized and subsequently inoculated with viable 4T1 murine breast cancer cells.

In summary, reduced gold nanoparticles derived from tumor lysate enhance antigen presentation, maximizing the immune response from innate and adaptive immune systems and inhibiting tumor implantation in a triple-negative breast cancer murine model, preventing tumoral recurrence. Further studies should be performed with other cell lines to validate this nanosystem.

## Figures and Tables

**Table 1:** Size, ζ potential, and protein concentration in AuLtNPs.

Sampling Day	SPR Abs	Size nm	ζ Potential mV	Protein Concentration mg/mL
1	0.635	20.1 ± 5.4	−18.9	0.6126
15	0.865	36.7 ± 10.4	−21.4	0.754
30	1.21	77.3 ± 12.8	−27.6	0.902

**Table 2:** Pearson correlation among PRS, size, ζ potential, and protein concentration in AuLtNPs.

Correlation Pearson r	SPR Abs	Size nm	ζ Potential mV	Protein Concentration mg/mL
SPR	1.000	0.993	−0.993	0.995
Size	0.993	1.000	−1.000	0.975
ζ potential	−0.993	−1.000	1.000	−0.976
Protein concentration	0.995	0.975	−0.976	1.000

**Table 3:** Survival analysis of experimental groups.

Experimental Group	Survival Analysis
	Days (%)
Control	21 (100%)
Tumor cell lysate (LT)	21 (100%)
CsNps	18 (100%)
AuLtNps	90 (100%)
LT+Adjuvant	21 (33.3%), 8 (66.6%)
CsNps+LT	16 (66.6%), 28 (33.3%)

## Data Availability

The raw data supporting the conclusions of this article will be made available by the authors on request.
